# 1086. COVID-19 in a Comprehensive Cancer Center: 2020-2022

**DOI:** 10.1093/ofid/ofac492.926

**Published:** 2022-12-15

**Authors:** Patricia Mulanovich, Roy F Chemaly, Bruno Granwehr, Kelly McConn, Physician Assistant, Nina Patel, Physician Assistant, Issam I Raad, Javier Adachi

**Affiliations:** MD Anderson, Houston, Texas; MD Anderson, Houston, Texas; MD Anderson, Houston, Texas; MD Anderson, Houston, Texas; MD Anderson, Houston, Texas; MD Anderson UT, Houston, Texas; MD Anderson Cancer Center, Houston, Texas

## Abstract

**Background:**

Patients with COVID-19 and underlying malignancies, particularly those receiving immunosuppressive therapy, are at higher risk of severe COVID-19 disease.

Our retrospective cohort study examines the outcomes of COVID-19 infection in patients with different underlying malignancies admitted to a 710- beds comprehensive cancer center during the first 2 years of the pandemic.

**Methods:**

All patients with cancer admitted to MD Anderson Cancer Center with a positive PCR test for SARS-CoV-2 were included in a clinical case registry from 3/22/20 (first hospitalized COVID-19 patient) to 3/31/22. This clinical registry was approved at the beginning of the COVID-19 pandemic by the Quality Improvement Assessment Board at MDACC. Clinical information including type of malignancy, date of admission, length of stay, need for invasive mechanical ventilation (IMV), and in-hospital mortality was obtained from their electronic medical records. Statistical analysis was performed using a two-proportion z-test where p< 0.05 was considered significant.

**Results:**

A total of 1748 patients with cancer and COVID-19 infection were admitted over a 2-year period (3.2% of total hospital admissions during the same period), 49% had hematological malignancies (HM) (see table). Patients with HM had significantly higher readmission rates (17.3% vs 9.1%, p< 0.0001), IMV rates (7.8% vs 4.4%, p=0.0029), and inpatient mortality rates (13.6% vs 7.1%, p< 0.0001). compared to patients with solid tumors (ST). Total mortality rate was 8.8% (154 patients), even higher in patients with different types of HM, such as lymphoma 18.1%, AML 14.2%, MM 8.4%, CML 7.1% while the mortality for ST was 7.1%.

COVID-19 Hospitalized Patients at UT-MDACC (3/22/20-3/31/22)

**Figure:**
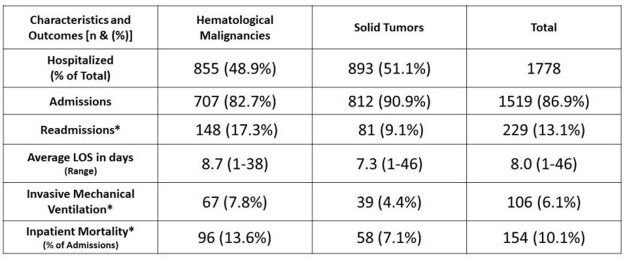

UT-MDACC: The University of Texas MD Anderson Cancer Center

*p-value <0.01 for z-test of 2 proportions (one-tailed)

**Conclusion:**

HM patients hospitalized with COVID-19 infection had more severe disease and worse outcomes based on readmissions, IMV, and mortality rates. Preventive measures, prompt diagnosis and early treatments should be considered on this patient population.

**Disclosures:**

**Roy F. Chemaly, MD/MPH**, Karius: Advisor/Consultant|Karius: Grant/Research Support.

